# Cross-Scale Synergistic Reinforcement of Ultra-High-Performance Concrete by Cellulose Nanofibers and Polyoxymethylene Fibers: Mechanical Behavior and Microstructural Mechanisms

**DOI:** 10.3390/ma19184005

**Published:** 2026-09-20

**Authors:** Peng Yu, Xueyan Han, Panfei Zheng, Xinghua Liu

**Affiliations:** 1 China Airport Planning & Design Institute Co., Ltd. Northwest Branch, Xi’an 710075, China; yupeng19910624@163.com; 2 Air Traffic Control and Navigation College, Air Force Engineering University, Xi’an 710043, China; 3 Civil Aviation Research Base (Beijing) Co., Ltd., Beijing 101300, China; chddominic@163.com; 4 School of Highway, Chang’an University, Xi’an 710064, China; liu617820@gmail.com

**Keywords:** ultra-high-performance concrete, polyoxymethylene fiber, cellulose nanofiber, flexural performance, dynamic mechanical properties, reinforcement mechanism

## Abstract

This study proposes a cross-scale reinforcement strategy for ultra-high-performance concrete (UHPC) using polyoxymethylene fibers (POMFs) and cellulose nanofibers (CNFs). The mechanical behavior and cross-scale synergistic reinforcement mechanisms of POMF/CNF-reinforced UHPC are systematically investigated through compressive, three-point bending, and split Hopkinson pressure bar (SHPB) tests, together with thermogravimetric (TG) analysis, scanning electron microscopy (SEM), and pore structure characterization. The results show that POMFs improve the post-cracking load-bearing capacity of UHPC through macroscopic crack bridging and pullout-induced energy dissipation, thereby transforming the failure mode from brittle to quasi-ductile. The incorporation of CNFs further enhances the matrix strength and flexural performance. At a CNF content of 0.2 wt.%, the compressive and flexural strengths of POMF-reinforced UHPC increase by 9.7% and 21.9%, respectively. Under high strain rates, the POMF-CNF composite system exhibits a pronounced strain-rate strengthening effect. In particular, UHPC containing 2 vol.% POMFs and 0.2 wt.% CNFs achieves both high dynamic load-bearing capacity and energy dissipation capacity, with an impact toughness of 5.2 MJ/m^3^ at a high strain rate. Microstructural results indicate that CNFs improve matrix compactness and resistance to microcracking by promoting hydration, refining the pore structure, and providing nanoscale bridging. They also enhance stress transfer across the POM fiber–matrix interface, which acts synergistically with the macroscopic crack-bridging effect of POMFs to produce cross-scale toughening. This study provides a reference for the cross-scale design of UHPC with high strength, high toughness, and superior resistance to dynamic damage.

## 1. Introduction

Ultra-high-performance concrete (UHPC) has broad application prospects in high-performance infrastructure and protective structures because of its high strength and excellent durability [1,2,3]. These superior properties primarily arise from its low water-to-binder ratio, optimized particle packing, and dense microstructure, which together form a high-strength load-bearing matrix [4,5,6]. However, the high density and strength of UHPC also result in pronounced brittleness [7]. Under dynamic loading, rapid crack initiation and unstable propagation further intensify damage localization, reducing the energy dissipation and impact resistance of the material [8,9,10]. Therefore, achieving a balance between high strength and high toughness while improving the damage tolerance of UHPC under dynamic loading remains a key challenge in its performance optimization.

Introducing polymer fibers into UHPC is an effective approach to improving its strength, toughness, and post-cracking load-bearing capacity [11,12]. Compared with steel fibers, polymer fibers offer advantages such as corrosion resistance, high deformability, and low cost [13,14,15]. Among them, polyoxymethylene fibers (POMFs) combine relatively high strength and toughness, and can effectively improve the tensile, flexural, and fracture properties of UHPC through crack bridging and interfacial stress transfer [16,17]. However, the toughening effect of POMFs mainly acts on cracks from the microscale to the macroscale, while their ability to regulate nanoscale defects and early crack initiation remains limited [18]. Since the failure of UHPC involves a cross-scale process extending from the evolution of nanoscale defects to the propagation of macroscopic cracks, reinforcement at a single scale is insufficient to effectively regulate the entire damage evolution process. Therefore, incorporating reinforcing components at different length scales to achieve hierarchical control of crack evolution provides a promising strategy for further improving the strength and toughness of UHPC [19,20,21]. Nanoscale reinforcements can suppress crack initiation by regulating the microstructure and early-stage defects [22,23], whereas macroscale fibers restrict crack propagation through crack bridging and pullout-induced energy dissipation [24,25]. Cellulose nanofibers (CNFs) possess a high aspect ratio, excellent mechanical properties, and abundant surface hydroxyl groups, which enable strong interfacial interactions with the cementitious matrix [26,27]. Previous studies show that CNFs refine the pore structure, improve matrix compactness, and suppress the initiation and propagation of microcracks through nanoscale filling, hydration promotion, and nanoscale bridging [28,29].

However, CNFs mainly contribute to microstructural refinement of the matrix and regulation of microcracks, while their contribution to macroscopic crack bridging and post-cracking load-bearing capacity remains relatively limited [15,30]. Therefore, combining nanoscale CNFs with macroscale POMFs is expected to enable hierarchical control of crack initiation, propagation, and instability through complementary reinforcement across different length scales, thereby improving the overall mechanical performance of UHPC. From an engineering perspective, POMF–CNF-reinforced UHPC has potential applications in structures requiring enhanced crack control, post-cracking capacity, and impact resistance, such as airport pavements, bridge deck components, and protective structures. Despite this potential, the cross-scale synergistic mechanism between POMFs and CNFs in UHPC has not yet been systematically clarified, as existing studies mainly focus on their individual reinforcement effects. In particular, under dynamic loading, the effects of POM/CNF hybrid reinforcement on strain-rate sensitivity, dynamic strength, deformation behavior, and energy dissipation characteristics remain insufficiently understood. Accordingly, clarifying the cross-scale toughening and dynamic energy dissipation mechanisms of POMF–CNF-reinforced UHPC is important for developing UHPC with high strength, high toughness, and superior impact resistance, and for supporting its future engineering applications.

This study develops a cross-scale reinforced UHPC incorporating POMFs and CNFs, with a focus on the effects of CNF addition on compressive properties, flexural behavior, and dynamic response at high strain rates. Microstructural morphology, pore structure, and hydration evolution are characterized to clarify the cross-scale synergistic reinforcement mechanism between POMFs and CNFs. The results provide a reference for the cross-scale design of UHPC with high toughness and superior impact resistance.

## 2. Materials and Methods

### 2.1. Raw Materials

**Figure 1:**
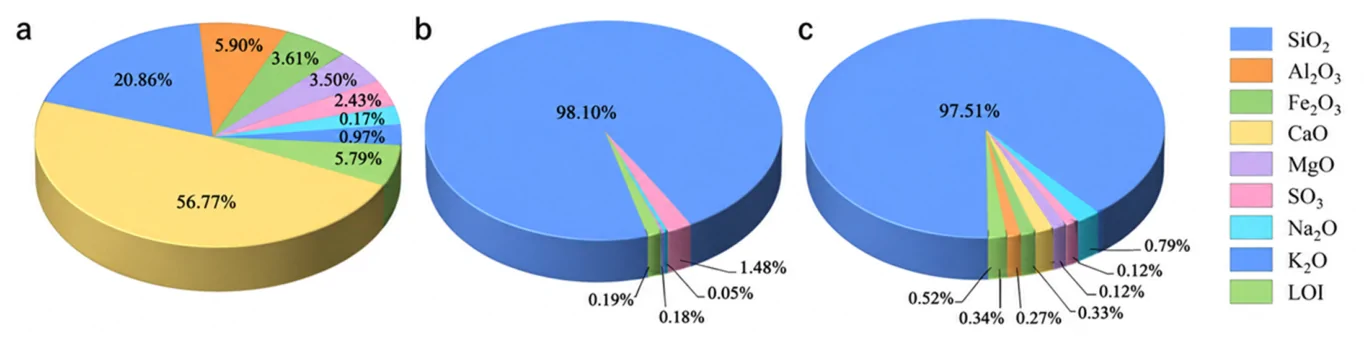
Chemical compositions (wt.%) of (**a**) cement, (**b**) silica fume, and (**c**) quartz sand.

Ordinary Portland cement (P.O. 52.5), supplied by Shandong Shanshui Cement Group Co., Ltd. (Jinan, China), and silica fume, produced by Henan Borun Foundry Materials Co., Ltd. (Zhengzhou, China), were used as the cementitious materials. Quartz sand with a particle size of 0.21–0.38 mm was used as the aggregate. The chemical compositions of these materials are shown in Figure 1. A polycarboxylate-based high-range water-reducing admixture with a water-reduction rate of 31% was added to improve the workability of the UHPC mixtures. Tap water was used for mixing.

POMF–CNF-reinforced UHPC was reinforced with two types of materials at different length scales: POMFs produced by Yuntianhua Group Co., Ltd. (Kunming, China), and CNFs supplied by Songhu Shenjian Technology Co., Ltd. (Dongguan, China). According to the manufacturer, the CNFs were derived from wood pulp and produced through mechanical processing. The CNFs were supplied as an aqueous suspension with a solid content of 2 wt.%, which facilitated their uniform dispersion in the UHPC matrix. The physical properties of the two reinforcing materials are summarized in Table 1.

The classic UHPC matrix developed by Richard and Cheyrezy [31] was adopted in this study. Six UHPC mixtures were designed, including four POMF–CNF-reinforced UHPC mixtures and two control mixtures. Based on previous studies [14,32] and our preliminary tests, the POMF volume fraction was fixed at 2% to provide a representative reinforcement level with satisfactory mechanical performance, while the CNF dosage was varied to investigate its cross-scale reinforcing effect. The water-to-binder ratio (W/B) was fixed at 0.18 for all mixtures, as shown in Table 2. The CNF dosages, calculated based on the dry mass of CNFs, were 0%, 0.05%, 0.1%, 0.2%, and 0.3% by mass of cement. The mixtures were designated according to the POMF volume fraction and CNF dosage. For example, P0C0 represents the plain UHPC matrix without POMFs or CNFs. P2C0 represents POMF-reinforced UHPC containing 2 vol% POMFs but no CNFs. P2C0.05 represents POMF–CNF-reinforced UHPC containing 2 vol% POMFs and 0.05 wt% CNFs by mass of cement.

### 2.2. Sample Preparation

**Figure 2:**
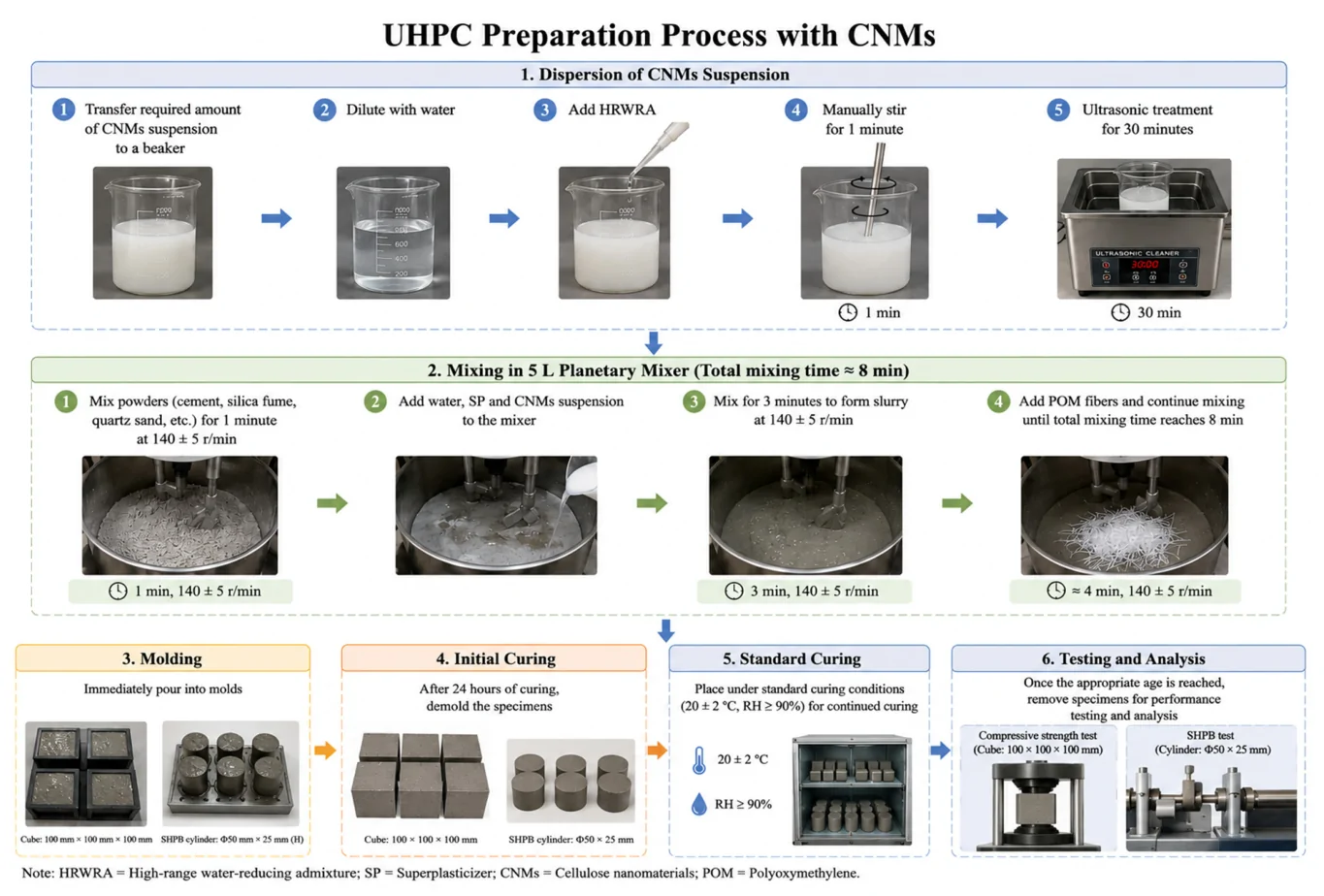
Preparation procedure of UHPC incorporating CNFs and POMFs.

The preparation procedure of UHPC reinforced with CNFs and polyoxymethylene fibers is illustrated in Figure 2. To achieve a homogeneous dispersion of CNFs, the required amount of CNF suspension was first transferred into a beaker and diluted with water. Subsequently, the high-range water-reducing admixture was introduced, followed by manual stirring for 1 min and ultrasonic treatment for 30 min to ensure adequate dispersion of the nanofibers. The mixing process was carried out using a 5 L planetary mixe (JJ-5, Wuxi Jianyi Instrument & Machinery Co., Ltd., Wuxi, China)r with a total mixing duration of approximately 8 min. Initially, all dry constituents were blended for 1 min at a rotational speed of 140 ± 5 r/min. Afterwards, water, superplasticizer, and the dispersed CNFs suspension were gradually added and mixed for 3 min at the same speed until a uniform cementitious slurry was obtained. Finally, the POMFs were gradually incorporated into the slurry, and mixing was continued until the total mixing time reached 8 min. Immediately after mixing, the fresh mixture was cast into the molds and sealed with plastic film to minimize moisture evaporation. After curing in the molds for 24 h, the specimens were demolded and transferred to a standard curing chamber maintained at 20 ± 2 °C and a relative humidity of no less than 90%. After curing for 28 days, the specimens were removed for subsequent mechanical testing and microstructural characterization.

### 2.3. Experimental Methods

The experimental program included quasi-static mechanical testing, dynamic mechanical testing, and microstructural characterization to systematically evaluate the mechanical response and underlying reinforcement mechanisms.

#### 2.3.1. Quasi-Static Mechanical Testing

The compression tests were conducted to evaluate the effect of different CNF dosages on the compressive strength of UHPC. Cubic specimens with dimensions of 100 × 100 × 100 mm^3^ were prepared. During testing, the compressive load was applied continuously at a loading rate of 1.2 MPa/s until specimen failure, as indicated by extensive cracking. The compressive strength was calculated by dividing the recorded peak load by the loaded cross-sectional area of the specimen. Three specimens were tested for each mixture, and the average value was reported as the final result.

Three-point bending tests were conducted to characterize the flexural behavior of UHPC. Prismatic specimens with dimensions of 40 × 40 × 160 mm^3^ were used, with a span length of 100 mm. The tests were performed under displacement control using a CMT 5105 universal testing machine (MTS Systems (China) Co., Ltd., Shenzhen, China). with a maximum load capacity of 100 kN. The loading rate was maintained at 0.5 mm/min. To quantitatively evaluate these changes, the flexural strength and toughness parameters were calculated according to JG/T 472–2015 [33]. This method evaluates flexural toughness at several prescribed midspan deflections defined as L/k, where L is the span length, and k takes values of 500, 300, 250, 200, and 150. However, UHPC exhibits pronounced deflection-hardening behavior and a relatively long nonlinear response. For k = 300 and 200, the corresponding L/k deflections of approximately 0.33 and 0.50 mm may still fall within the strain-hardening stage. Therefore, these deflection levels may not adequately characterize the flexural toughness of UHPC. To ensure that the elastic, strain-hardening, and strain-softening stages were all included in the toughness evaluation, k = 150 was selected in this study. After correcting the initial seating segment of the load–deflection curve, the equivalent initial flexural tensile strength (*f_e,p_*), initial flexural toughness ratio (*R_e,p_*), equivalent residual flexural tensile strength (*f_e,k_*), and residual flexural toughness ratio (*R_e,k_*) were calculated. The corresponding equations are given as follows:

(1) $$R_{e,p}=f_{e,p}/f_{m}$$

(2) $$f_{e,p}=\frac{\Omega_{p}L}{bh^{2}\delta_{p}}$$

where *R_e,p_* is the initial flexural toughness ratio; *f_e,p_* is the equivalent initial flexural tensile strength, in MPa; b is the specimen width, in mm; h is the specimen height, in mm; L is the span length, in mm; *δ_p_* is the midspan deflection corresponding to the peak load, in mm; Ω*_p_* is the area under the load–deflection curve up to *δ_p_*, in N·mm; and *f_m_* is the flexural tensile strength of the UHPC specimen, in MPa.

(3) $$R_{e,k}=f_{e,k}/f_{m}$$

(4) $$f_{e,k}=\frac{\Omega_{p,k}L}{bh^{2}(\delta_{k}-\delta_{p})}$$

where *R_e,k_* is the flexural toughness ratio corresponding to a prescribed midspan deflection *δ_k_*; fe,k is the equivalent residual flexural tensile strength corresponding to *δ_k_*, in MPa; Ω_p,k_ is the area under the load–deflection curve between *δ_p_* and *δ_k_*, in N·mm; *δ_p,k_* is the increase in midspan deflection from *δ_p_* to *δ_k_*, in mm; and *δ_k_* is the prescribed midspan deflection defined as L/k, in mm.

#### 2.3.2. Dynamic Mechanical Testing

**Figure 3:**
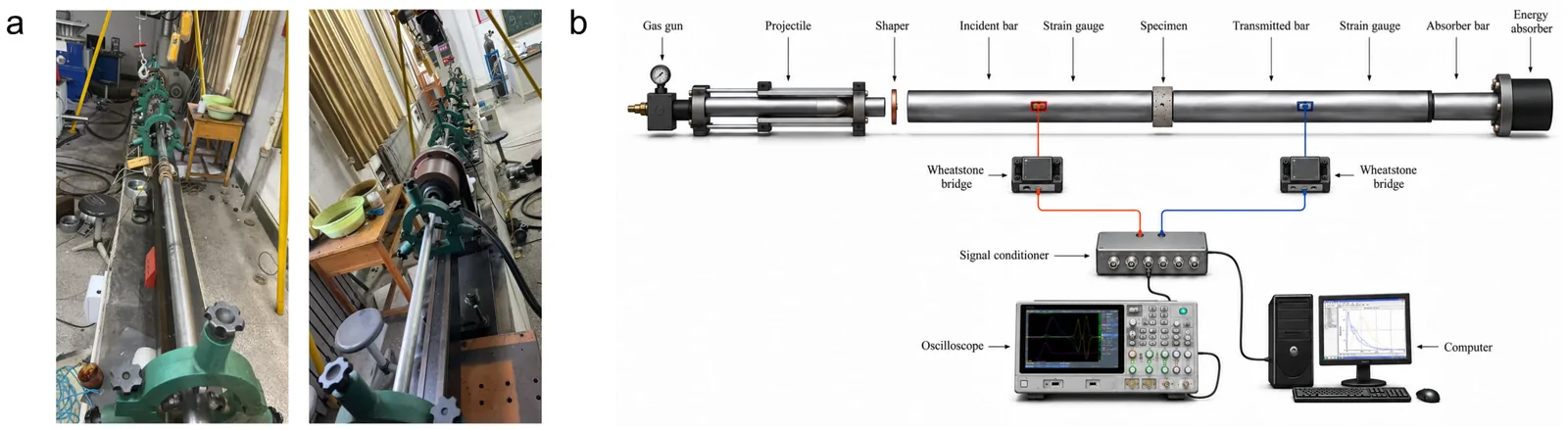
SHPB test system: (**a**) experimental setup, (**b**) schematic diagram.

A split Hopkinson pressure bar (SHPB) system (Luoyang Liwei Technology Co., Ltd., Luoyang, China) is used to evaluate the dynamic mechanical properties of UHPC reinforced with CNFs and POMFs, as shown in Figure 3. Cylindrical specimens with a diameter of 50 mm and a height of 25 mm are used. After fine grinding, the two end surfaces are aligned coaxially between the incident and transmission bars, and a thin layer of lubricant is applied to minimize interfacial friction. A thin copper disk is placed at the end of the incident bar as a pulse shaper to improve the incident waveform and facilitate dynamic stress equilibrium within the specimen before failure. Impact pressures of 0.2, 0.3, and 0.4 MPa are applied, and three replicate specimens are tested for each mixture at each pressure level.

The incident, reflected, and transmitted waves are measured using strain gauges attached to the pressure bars and recorded by a signal conditioning system and an oscilloscope. A test is considered valid when the force–time histories at the two specimen ends approximately overlap. Based on one-dimensional stress wave theory, the dynamic stress, strain, and strain rate are calculated using the three-wave method [34], and the dynamic compressive strength, peak strain, and dynamic impact toughness are obtained from the dynamic stress–strain curves.

The dynamic compressive strength *f*_cd_ is defined as the maximum stress on the dynamic stress–strain curve, and the peak strain *ε_p_* is the corresponding strain at *f*_cd_. The dynamic increase factor DIF is used to evaluate the strain-rate strengthening effect of UHPC and is calculated as follows:

(5) $$DIF=\frac{f_{cd}}{f_{cs}}$$

where *f_cd_* and *f_cs_* denote the dynamic and quasi-static compressive strengths, respectively.

Dynamic toughness is characterized by the area under the dynamic stress–strain curve and is calculated as follows:

(6) $$T_{d}=\int_{0}^{\varepsilon_{u}}\sigma (\varepsilon )d\varepsilon$$

#### 2.3.3. Microstructural Characterization

1. Thermogravimetric analysis

Thermogravimetric analysis (TGA) was performed using a SDT 650 simultaneous thermal analyzer (TA Instruments, New Castle, DE, USA) to characterize the hydration products in the UHPC paste. Fragments of the fractured paste specimens were collected and immersed in isopropanol for 3 days to terminate hydration. The dried samples were then ground and passed through a 200-mesh sieve. The test was conducted under a nitrogen atmosphere. The temperature was increased from 35 to 500 °C at a heating rate of 10 °C/min. Calcium silicate hydrate (C-S-H) is the primary binding phase in hydrated cement and plays a key role in matrix densification and strength development. Therefore, its content was quantified to evaluate the effect of CNFs on the formation of hydration products in the UHPC paste. According to Ref. [35], the C-S-H content was calculated using the following equation:

(7) $$C\text{-}S\text{-}H\;(\%)=\frac{M_{\mathrm{CSH}}}{2.1M_{H}}\times \Delta m_{\mathrm{CSH}}\;(\%)$$

where *M*_H_ and *M*_CSH_ are the molecular weights of water and C-S-H, respectively, and $\Delta m_{\mathrm{CSH}}$ is the mass loss between 105 and 400 °C.

2. Scanning electron microscopy

Small cubic specimens with dimensions of 10 × 10 × 10 mm were extracted from the fractured specimens after the impact tests. The microstructure was examined using a scanning electron microscope (SEM, Carl Zeiss Microscopy GmbH, Jena, Germany) at an accelerating voltage of 15 kV. Secondary electron imaging was used to characterize the distribution of CNFs and hydration products within the UHPC matrix.

3. Air-void structure characterization

**Figure 4:**
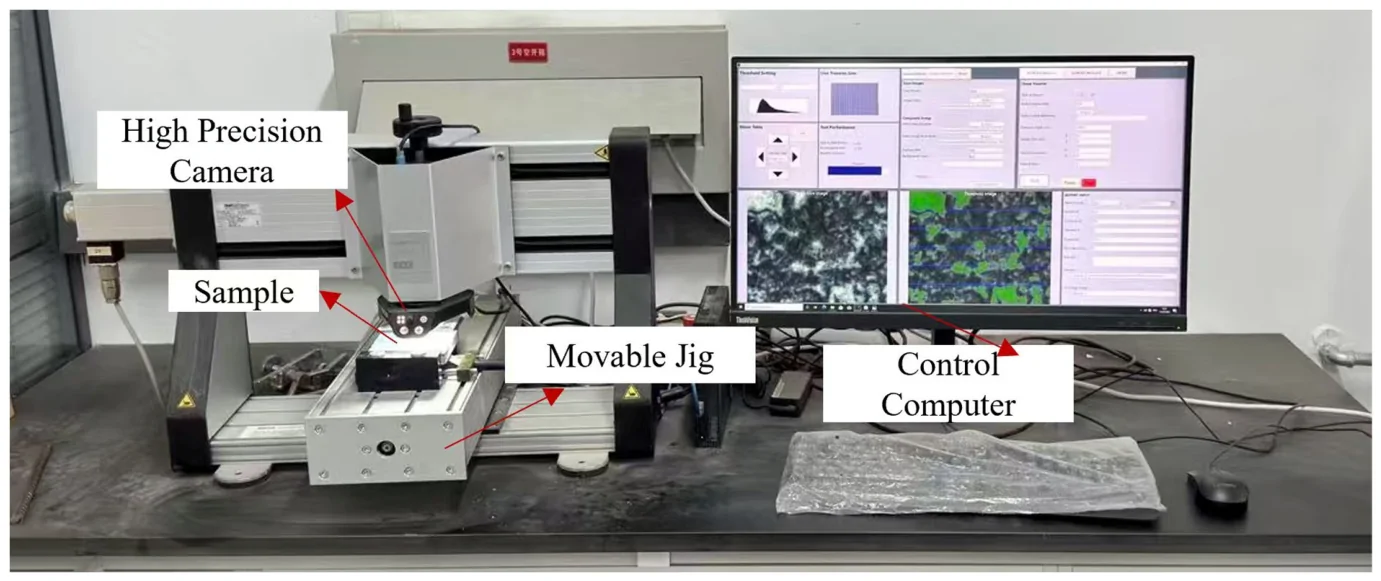
RapidAir 457-type hardened concrete air void structure analyzer.

To characterize the pore structure of UHPC incorporating cellulose nanofibers, a RapidAir 457 automated air-void analysis system (Germann Instruments A/S, Copenhagen, Denmark) was employed to evaluate the pore size distribution, air-void content, and specific surface area. Before testing, UHPC specimens with dimensions of 100 mm × 100 mm × 20 mm were successively polished using 80-, 320-, 600-, 1000-, and 1500-grit sandpapers until a smooth, scratch-free surface was obtained. The polished surface was then coated with carbon black ink and allowed to dry naturally. Subsequently, nano-calcium carbonate powder was uniformly spread over the specimen surface to fill the air voids, thereby enhancing the contrast between the voids and the surrounding cementitious matrix for image acquisition. The air-void characteristics were analyzed in accordance with ASTM C457/C457M [36]. Based on the number of air voids intersected and the cumulative traverse length obtained from a fixed scanning path, the air content and air-void size distribution of the hardened UHPC were determined. The experimental setup for the air-void analysis is presented in Figure 4.

## 3. Results and Discussion

This section discusses the effects of POM fibers and CNFs on the compressive, flexural, and dynamic mechanical properties of UHPC, followed by an analysis of the underlying reinforcement mechanisms.

### 3.1. Compressive Strength

**Figure 5:**
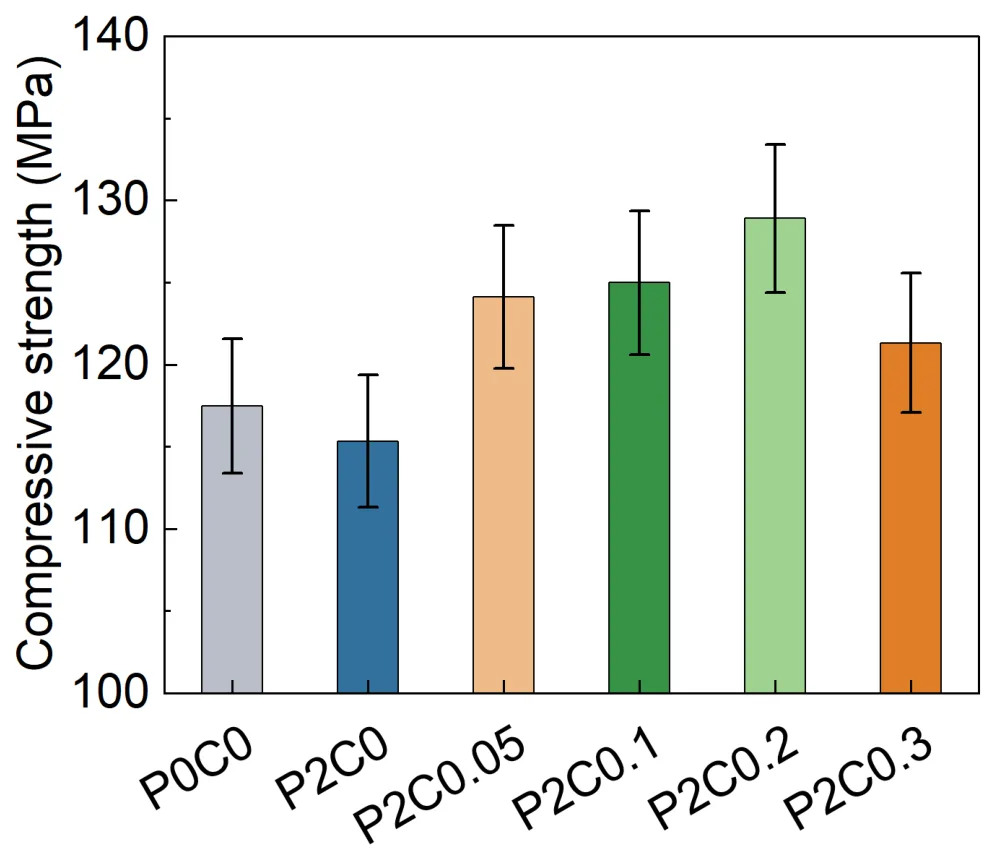
Effect of CNFs content on the compressive strength of POMFs-reinforced UHPC.

As shown in Figure 5, the incorporation of POMFs alone slightly reduced the compressive strength of UHPC. The compressive strength decreased from 117.47 MPa for P0C0 to 115.33 MPa for P2C0, which can be attributed to the dilution effect of low-modulus polymer fibers and the introduction of additional fiber–matrix interfaces [18]. However, after incorporating CNFs, the compressive strength exhibited an improvement and followed a typical initial enhancement followed by a deterioration trend with increasing CNF content. The maximum compressive strength of 128.89 MPa was achieved at 0.2 wt% CNF, representing a 9.7% increase compared with the reference UHPC. This improvement can be mainly attributed to the nano-filling effect of CNFs, their promotion of hydration reactions, and the refinement of the interfacial transition zone [23,37]. When the CNF content increased to 0.3 wt%, the compressive strength decreased, which was mainly related to CNF agglomeration and increased water demand caused by the excessive specific surface area.

### 3.2. Flexural Performance

The flexural performance was evaluated in terms of load–deflection behavior, flexural strength, and flexural toughness to clarify the effects of POM fibers and CNFs on the bending response of UHPC.

#### 3.2.1. Flexural Load–Deflection Curve

**Figure 6:**
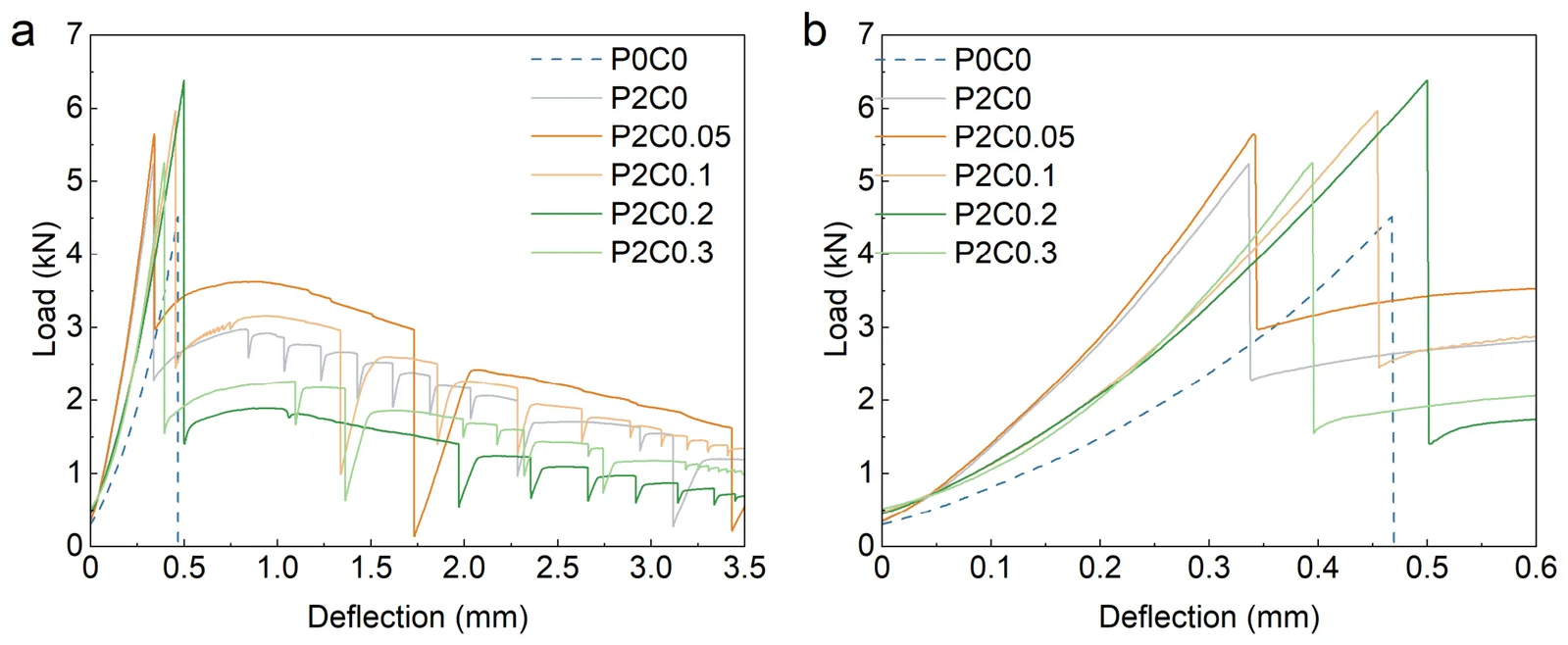
Flexural load–deflection curves of UHPC specimens: flexural load–deflection curves for specimens with deflections of (**a**) 0–3.5 mm and (**b**) 0–0.6 mm.

The load–deflection curves of POMF-reinforced UHPC with different CNF dosages are shown in Figure 6. Compared with the UHPC matrix, the addition of 2 vol% POMFs changed the load–deflection response. For P0C0, the load dropped sharply after reaching the peak, indicating typical brittle failure. In contrast, P2C0 exhibited a much higher peak load and a more gradual post-peak decline. This result indicates that POMFs substantially improved the post-cracking load-carrying capacity of UHPC and promoted a transition from brittle fracture toward quasi-ductile failure. This improvement can be mainly attributed to the crack-bridging effect of POMFs. The fibers bridge developing cracks and continuously transfer tensile stress during crack propagation. As a result, the formation of a dominant through-crack is delayed, which improves the deformation capacity and energy absorption of the material.

The load–deflection response changed further after the incorporation of CNFs. As the CNFs dosage increased from 0 to 0.2%, the peak load increased progressively, with P2C0.2 reaching the highest value. This trend indicates that CNFs further enhanced the flexural load-carrying capacity of the UHPC matrix. However, the post-peak responses varied among the mixtures. Compared with P2C0, P2C0.05 showed a more gradual post-peak load reduction, suggesting that a moderate CNFs dosage further improved the post-cracking deformation capacity. When the CNFs dosage increased to 0.1% and 0.2%, the post-peak load decreased more rapidly. In particular, although P2C0.2 achieved the highest peak load, it exhibited the most pronounced post-peak softening. This behavior suggests that the matrix-strengthening effect of CNFs gradually became more dominant than the post-cracking energy-dissipation contribution of POMFs. When the CNFs dosage was further increased to 0.3%, the peak load decreased slightly, while the post-peak decline became less steep. This indicates a partial recovery of the post-cracking deformation capacity.

#### 3.2.2. Flexural Strength

**Figure 7:**
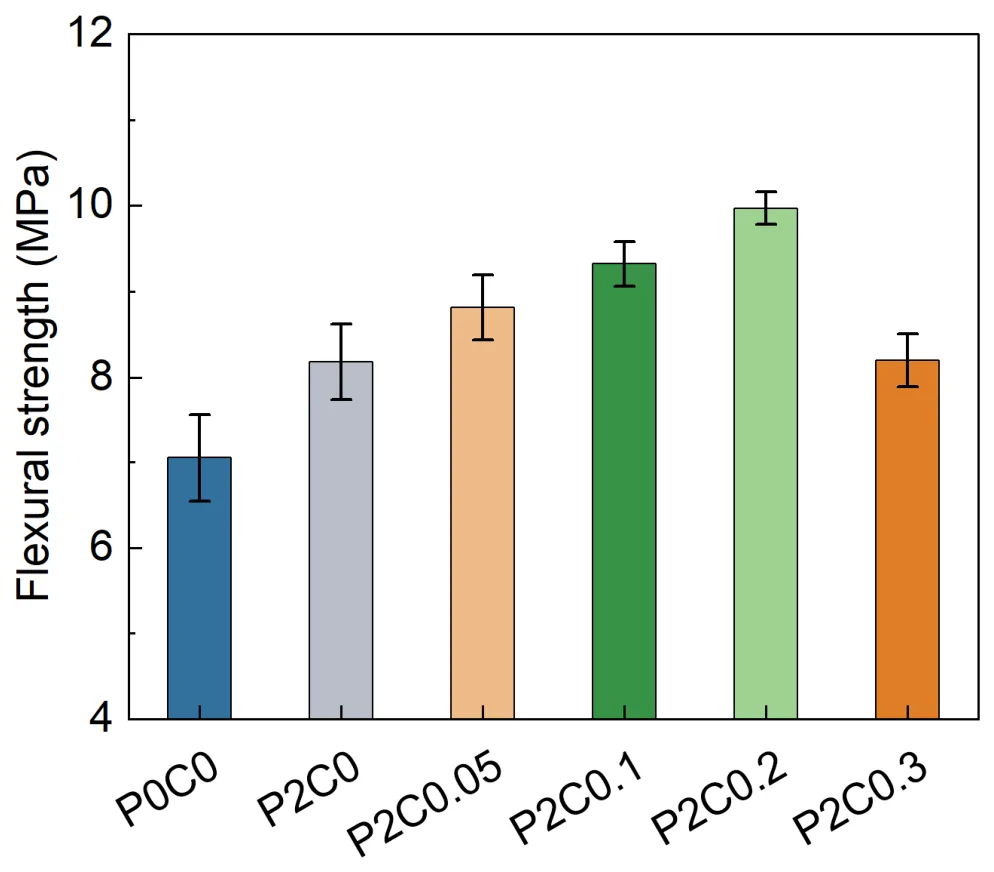
Flexural strength for POMF–CNF-reinforced UHPC.

The calculated flexural strengths of all mixtures are presented in Figure 7. The flexural strength showed a trend different from that of the compressive strength. After the incorporation of 2 vol% POMFs, the flexural strength increased by approximately 15.9%. This improvement can be attributed to the crack-bridging effect of POMFs. The fibers bridge microcracks, redistribute stresses near crack tips, and delay unstable crack propagation. These mechanisms allow a larger portion of the matrix to contribute to load transfer, thereby improving the flexural resistance of UHPC.

With the further incorporation of CNFs into POMF-reinforced UHPC, the flexural strength increased continuously as the CNF dosage increased. The maximum value of 9.97 MPa was obtained at a CNF dosage of 0.2%, representing a further increase of approximately 21.9% compared with P2C0. This trend was consistent with the compressive strength results. The improvement can be attributed to several effects of CNFs. CNFs can promote cement hydration and increase the formation of C-S-H gel. They can also refine the pore structure through a nanoscale filling effect and form nanoscale bridging networks among hydration products, thereby improving the continuity of the matrix [23,37]. Therefore, CNFs enhance the UHPC matrix through hydration promotion, pore refinement, and microstructural strengthening. These effects can also improve the interfacial bond between POMFs and the surrounding matrix, resulting in a higher flexural load-carrying capacity of POMF–CNF-reinforced UHPC. When the CNF dosage increased to 0.3%, the flexural strength decreased slightly. This reduction was mainly attributed to the local agglomeration of excessive CNFs [23]. Agglomeration reduces the uniform dispersion of CNFs within the cementitious matrix and weakens their hydration-promoting and nanoscale filling effects. As a result, the degree of matrix densification decreases, leading to a slight reduction in flexural strength.

#### 3.2.3. Flexural Toughness

**Figure 8:**
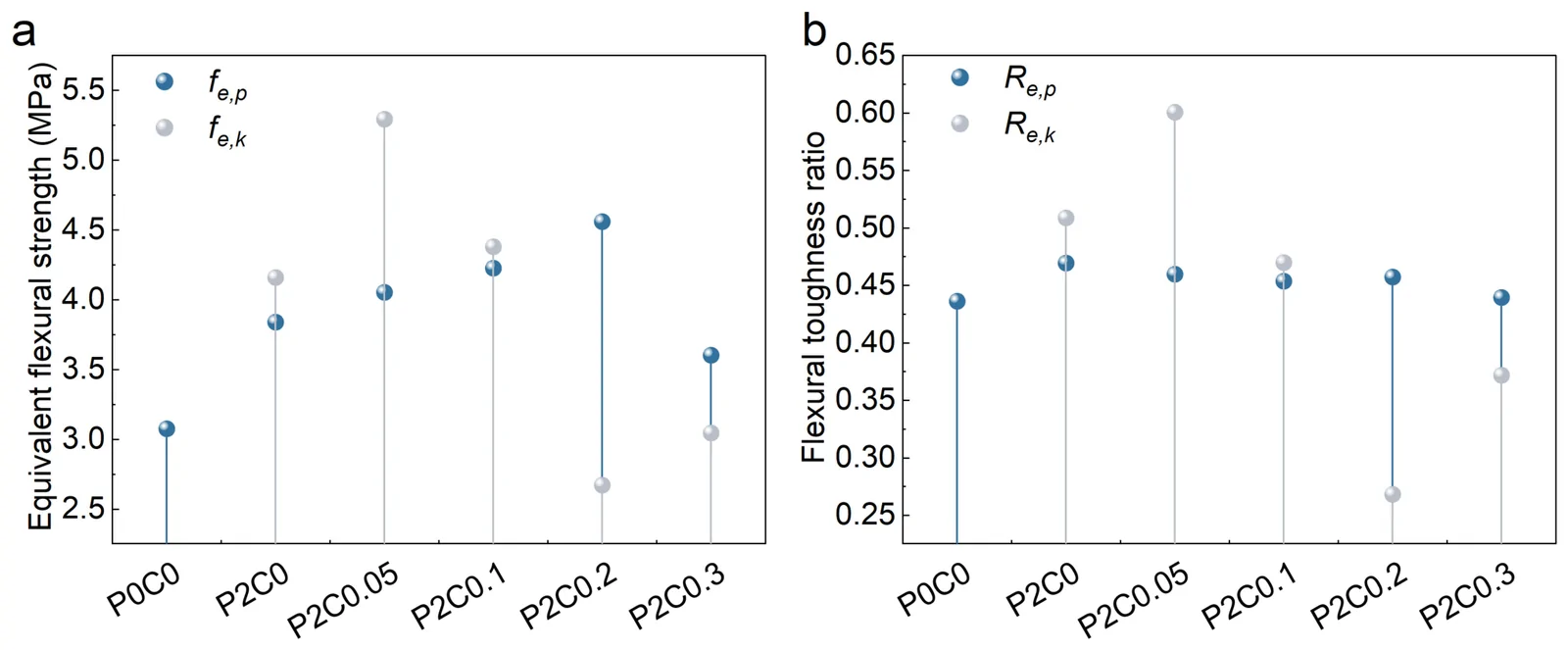
Equivalent flexural strength and flexural toughness ratio of UHPC with different CNF contents. (**a**) equivalent flexural strengths; (**b**) flexural toughness ratios.

Based on the evaluation method specified in JG/T 472-2015 [33], the load–deflection curves were first corrected for the initial seating segment. The equivalent initial flexural tensile strength (*f_e,p_*), initial flexural toughness ratio (*R_e,p_*), equivalent residual flexural tensile strength (*f_e,k_*), and residual flexural toughness ratio (*R_e,k_*) were then calculated. The results are shown in Figure 8. Compared with P0C0, the incorporation of 2% POMFs increases the equivalent initial flexural strength and initial flexural toughness ratio by 24.7% and 7.8%, respectively, indicating that POMFs enhance the pre-peak load-bearing and energy absorption capacities through crack bridging and stress redistribution. CNFs have a more pronounced effect on the residual flexural performance. When the CNF content increases to 0.05%, the equivalent residual flexural strength and residual flexural toughness ratio increase by 27.2% and 18.1%, respectively. This indicates that an appropriate CNF content strengthens the matrix and fiber–matrix interface, thereby promoting stable bridging and pullout-induced energy dissipation of POMFs. However, as the CNF content further increases to 0.1% and 0.2%, the flexural strength continues to increase, whereas the residual toughness decreases. P2C0.2 exhibits the highest flexural strength but the lowest residual flexural toughness ratio of 0.268. This result suggests that excessive interfacial strengthening, although beneficial to peak load capacity, restricts the interfacial slip and pullout of POM fibers and thus reduces post-cracking energy dissipation. When the CNF content increases to 0.3%, the residual flexural toughness ratio recovers to 0.372, which may be attributed to CNF agglomeration that weakens the interfacial strengthening effect.

### 3.3. Dynamic Mechanical Properties

The dynamic mechanical response was evaluated based on dynamic compressive strength, the dynamic increase factor (DIF), and impact toughness under different impact loading conditions.

#### 3.3.1. Dynamic Compressive Strength

**Figure 9:**
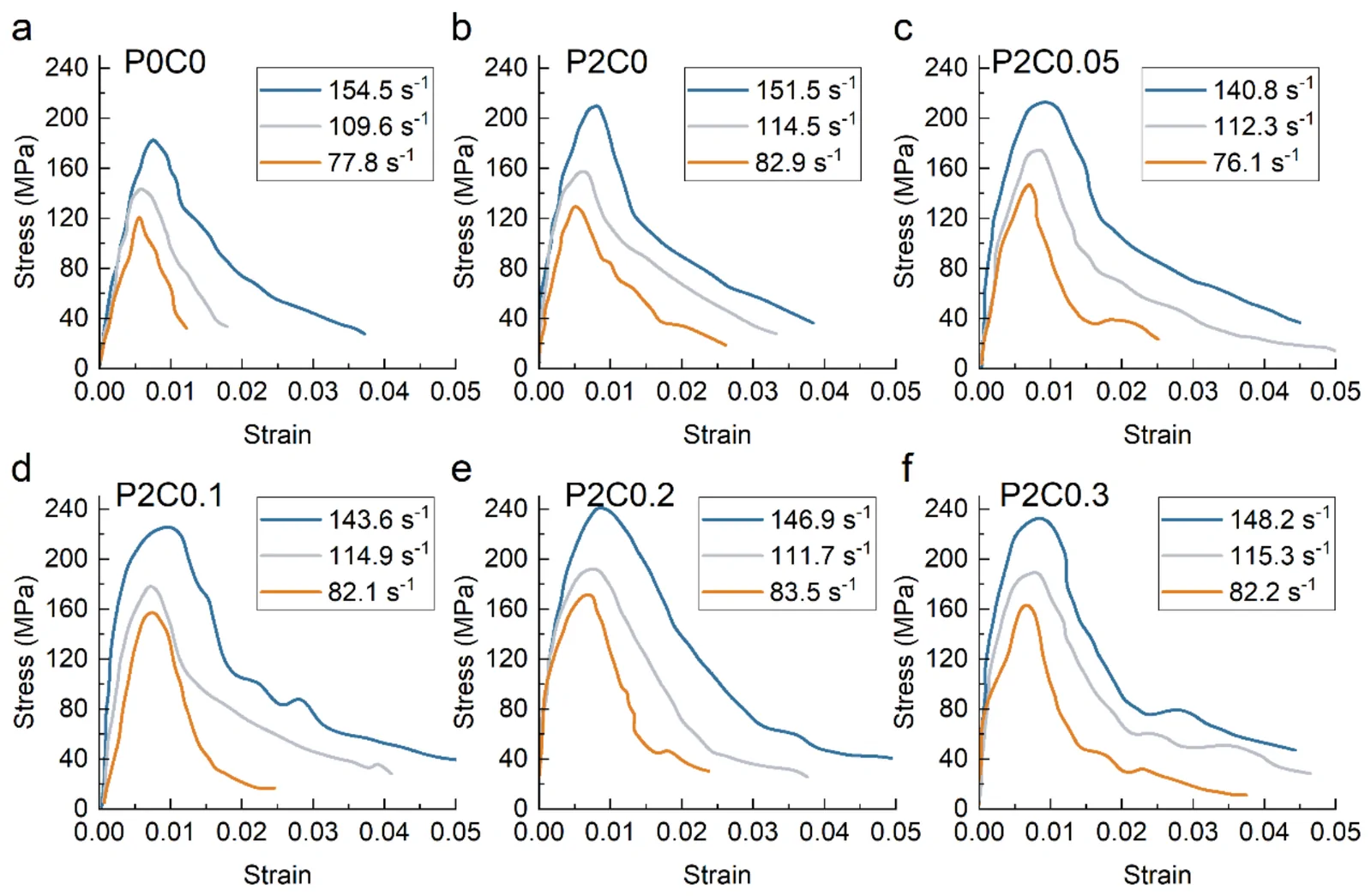
The stress–strain curves of UHPCs at different strain rates. (**a**) P0C0; (**b**) P2C0; (**c**) P2C0.05; (**d**) P2C0.1; (**e**) P2C0.2; (**f**) P2C0.3.

**Figure 10:**
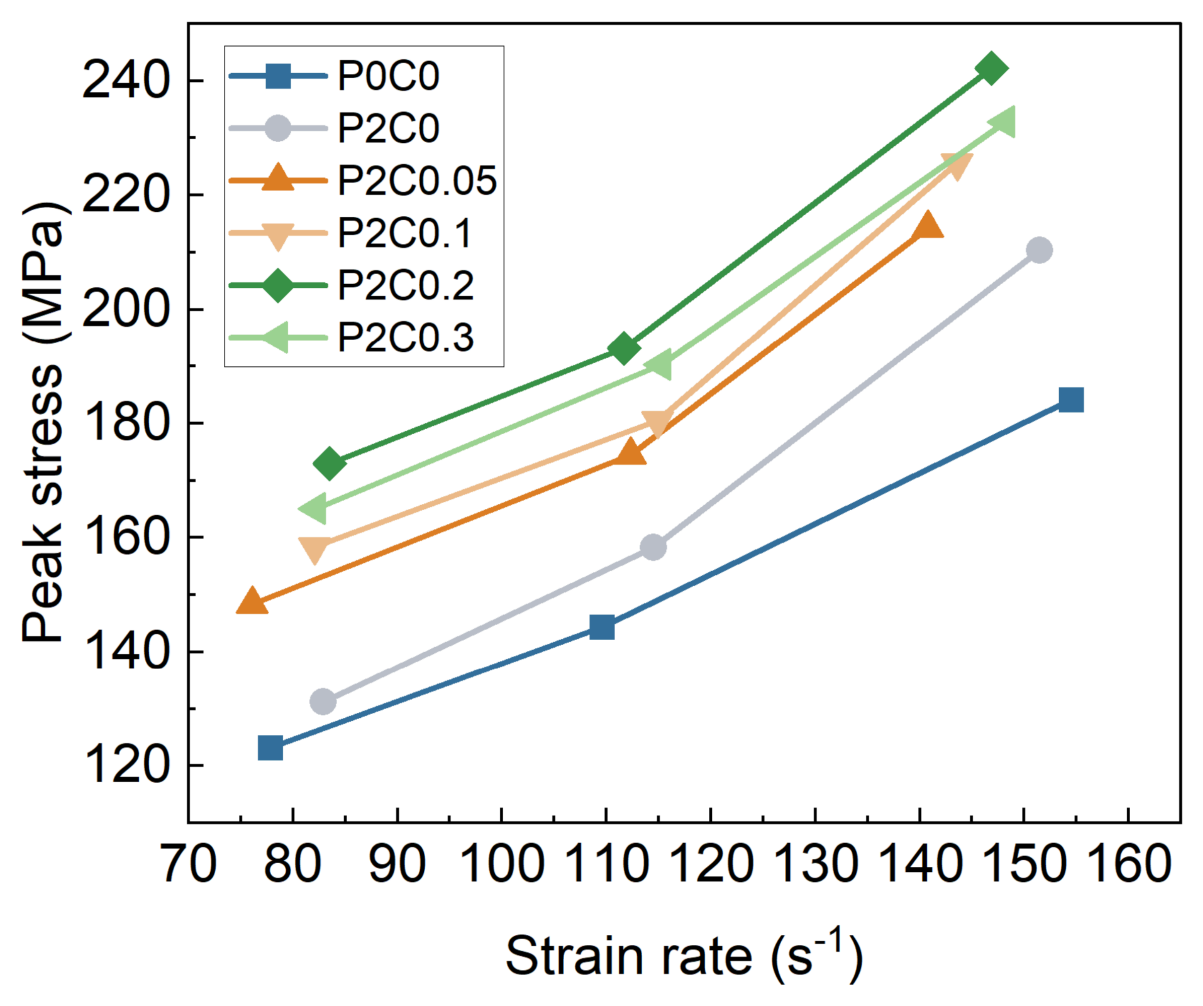
The peak stress of UHPCs at different strain rates.

Figure 9 presents the averaged dynamic compressive stress–strain curves of UHPC with different POMF and CNF dosages at various strain rates, based on three repeated SHPB tests. Overall, all mixtures exhibited similar dynamic compressive responses. The stress increased rapidly with strain and then entered a distinct strain-softening stage after reaching the peak. As the strain rate increased, the curves shifted upward, and the peak stress increased. At higher strain rates, the post-peak curves generally showed a longer descending branch. These results indicate that the investigated UHPC exhibited pronounced strain-rate sensitivity. Figure 10 further shows the peak stresses of UHPC with different POMF and CNF dosages at various strain rates. The peak stress increased continuously with increasing strain rate. For P0C0, the peak stress increased from approximately 123 to 183 MPa. After the incorporation of 2 vol% POMFs, the peak stress of P2C0 increased to approximately 131–210 MPa, indicating that POMFs improved the dynamic load-carrying capacity of UHPC. With the further incorporation of CNFs, the peak stress generally increased. Among all mixtures, P2C0.2 exhibited the highest peak stresses at all three strain-rate levels, reaching approximately 173, 193, and 242 MPa. When the CNF dosage was further increased to 0.3%, the peak stress decreased. These results demonstrate a clear dosage-dependent effect of CNFs on the dynamic strengthening of POMF-reinforced UHPC. An appropriate CNF dosage enhanced the dynamic strength, whereas excessive CNFs reduced the reinforcing efficiency.

The observed behavior can be attributed to the cross-scale synergistic effect of POMFs and CNFs. Under high strain-rate loading, crack propagation is constrained by the limited loading duration, while inertial effects become more pronounced. As a result, UHPC develops a higher dynamic strength. This trend is consistent with previous SHPB studies on fiber-reinforced UHPC [30,38]. At the macroscale, POMFs bridge cracks and restrain crack propagation, thereby improving crack resistance. At the nanoscale, well-dispersed CNFs can fill pores, densify the matrix, and improve the fiber–matrix interface. These effects enhance stress-transfer efficiency within the composite. Therefore, P2C0.2 exhibited the highest peak stress. The reduction in the peak stress of P2C0.3 may be associated with CNFs agglomeration, increased local porosity, or poorer dispersion at a higher CNFs dosage. This nonmonotonic dosage effect is also consistent with previous studies on CNFs-modified cementitious materials [27].

#### 3.3.2. Dynamic Increase Factor (DIF)

**Figure 11:**
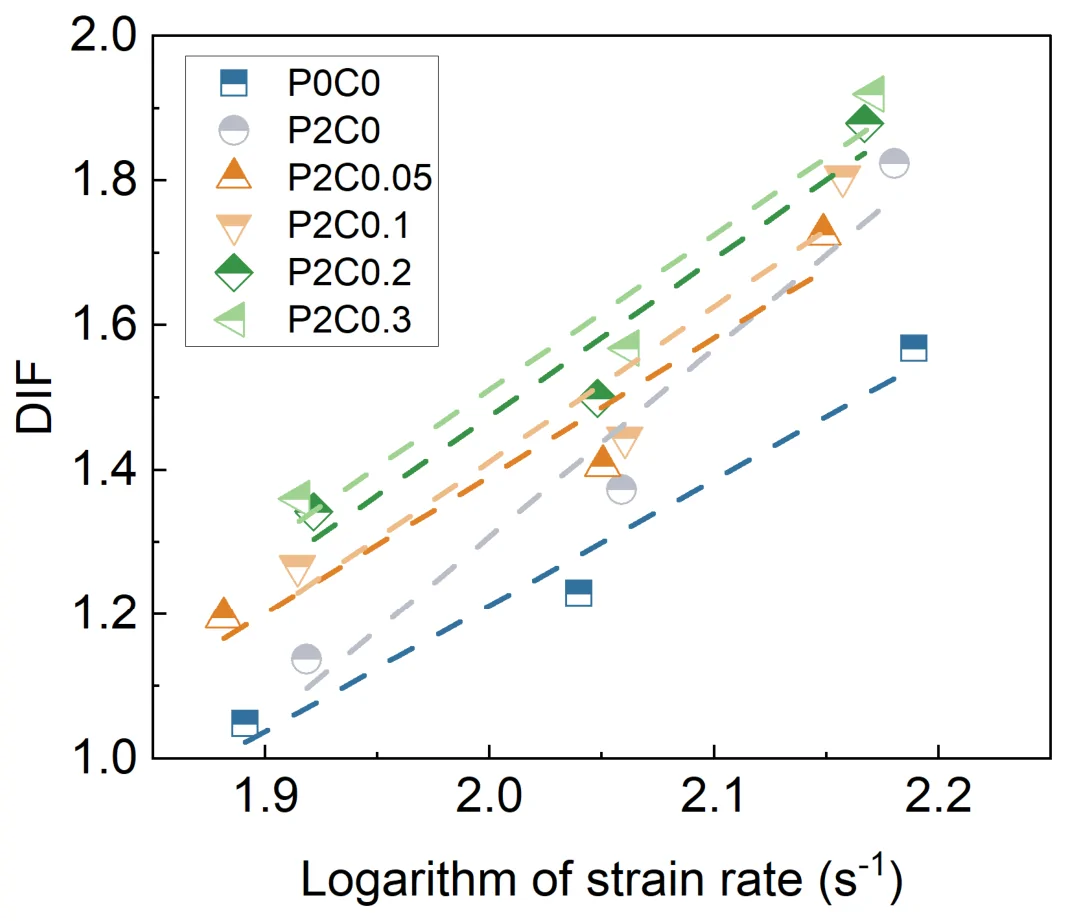
DIF of UHPC mixtures at different strain rates.

Figure 11 shows the variation in the DIF of UHPC with different POMFs and CNFs dosages at various strain rates. For all mixtures, the DIF increased approximately linearly with the logarithm of strain rate, indicating pronounced strain-rate sensitivity. After the incorporation of POMFs, P2C0 exhibited consistently higher DIF values than P0C0. The addition of CNFs further increased the DIF. This enhancement was most pronounced for P2C0.2 and P2C0.3, with DIF values approaching 1.9 at the highest strain rate. These results indicate that the combined incorporation of POMFs and CNFs enhances the dynamic strengthening effect of UHPC. However, the additional improvement became limited as the CNFs dosage increased further.

#### 3.3.3. Impact Toughness

**Figure 12:**
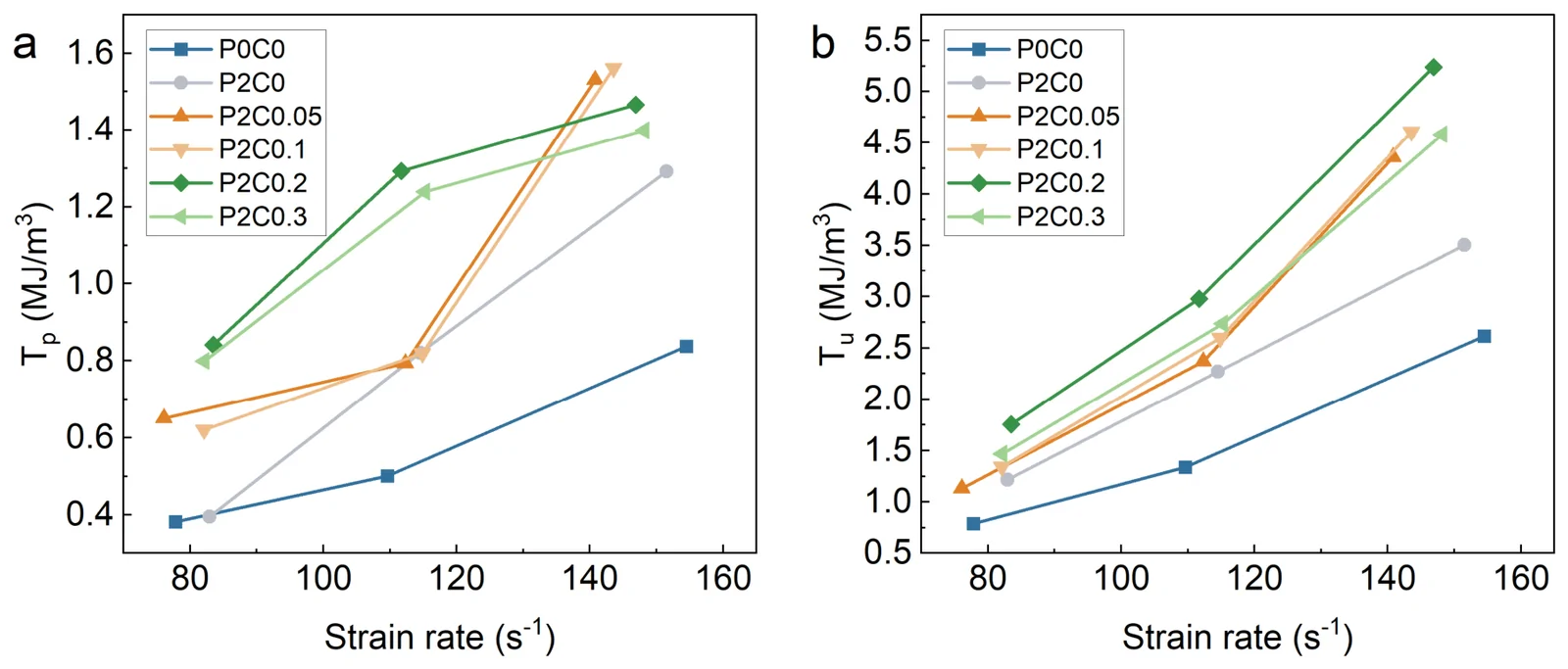
The impact toughness of UHPCs at different strain rates. (**a**) pre-peak toughness, *T*_p_; (**b**) total toughness, *T*_u_.

Figure 12 presents the impact toughness of UHPC at different strain rates. The pre-peak toughness (*T_p_*) was calculated as the area under the stress–strain curve from the onset of loading to the peak strain. This parameter represents the energy absorption capacity of the material before the peak stress is reached. The total toughness (*T_u_*) was calculated as the area under the stress–strain curve from the onset of loading to the post-peak strain corresponding to a residual stress of 60 MPa. It therefore characterizes the overall energy dissipation capacity during both the pre-peak loading stage and the post-peak damage stage. Overall, both *T_p_* and *T_u_* increased with increasing strain rate, indicating a pronounced strain-rate dependence of the energy absorption capacity of UHPC. Among all mixtures, P0C0 exhibited the lowest toughness. After the incorporation of POMFs, the increase in Tu was particularly pronounced. This result suggests that the toughening effect of POMFs mainly arises from post-peak energy-dissipation mechanisms, including crack bridging, fiber pullout, and frictional sliding. The incorporation of CNFs further improved the impact toughness. P2C0.2 exhibited the highest overall toughness, indicating that an appropriate CNFs dosage can enhance matrix densification and improve the fiber–matrix interface. These effects allow POMFs to provide more effective crack bridging and energy dissipation. However, the improvement in toughness did not increase monotonically with CNFs dosage. P2C0.2 showed the best performance in terms of Tu, reaching approximately 5.2 MJ/m^3^ at the highest strain rate. The increase in total toughness was more pronounced than that in pre-peak toughness. This indicates that the main advantage of POMF–CNF-reinforced UHPC lies in its enhanced post-peak crack-bridging and energy-dissipation capacity. When the CNFs dosage was further increased to 0.3%, Tu decreased. This result suggests that excessive CNFs may weaken the synergistic interaction at the fiber–matrix interface. Therefore, P2C0.2 provided a more favorable balance among matrix strengthening, interfacial bonding, and energy dissipation through POMFs pullout.

### 3.4. Cross-Scale Reinforcement Mechanism

To elucidate the cross-scale reinforcement mechanism of POM fibers and CNFs, hydration products, microstructural morphology, pore structure, and their correlations with mechanical properties were further analyzed.

#### 3.4.1. Hydration Product Analysis

**Figure 13:**
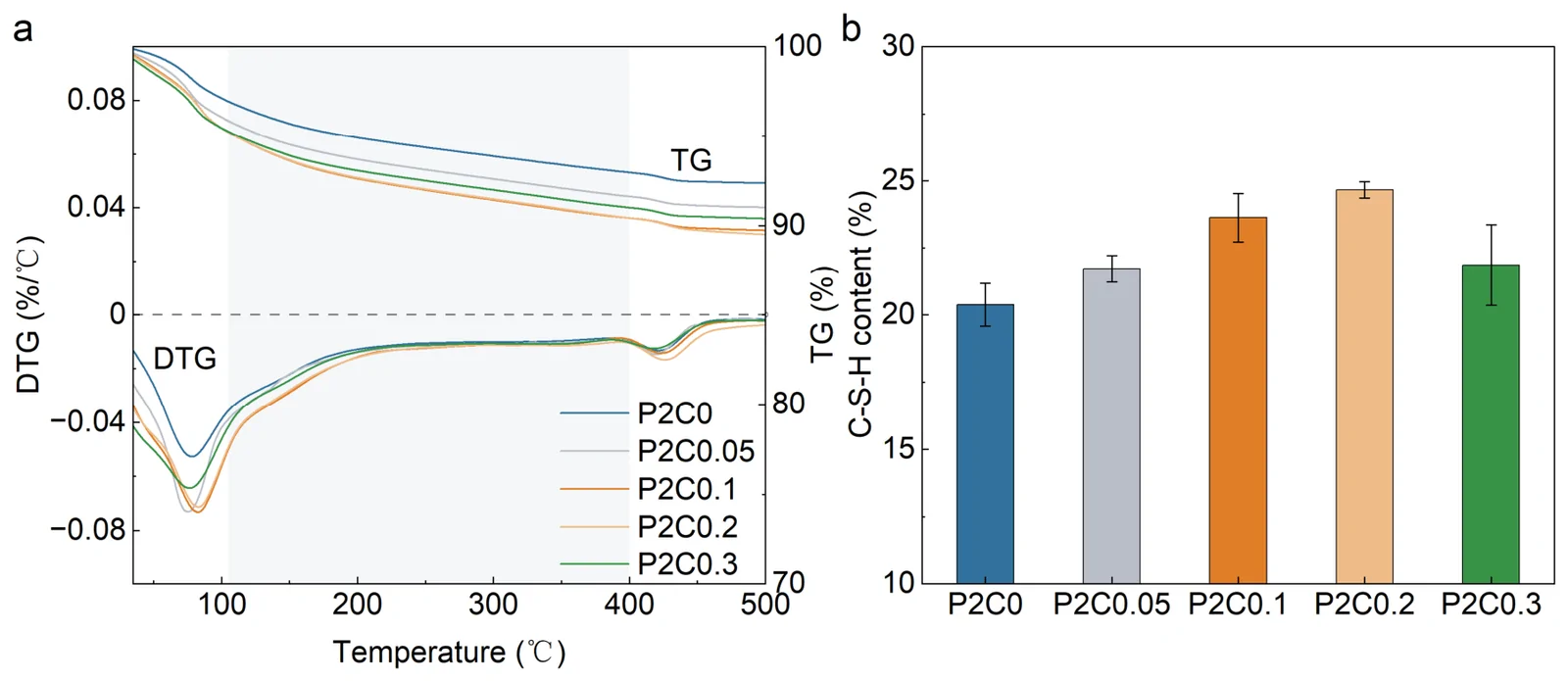
(**a**) DTG and TG curves; (**b**) Amount of C-S-H.

As shown in Figure 13, the thermogravimetry (TG) and derivative thermogravimetry (DTG) analyses were performed to investigate the effect of different CNF dosages on the formation of hydration products in POMF-reinforced UHPC. As shown in Figure 13a, the TG and DTG curves of all mixtures exhibited similar overall trends. This indicates that the incorporation of CNFs did not alter the main types of hydration products in the UHPC matrix. However, CNFs noticeably affected the extent of mass loss within different temperature ranges. Two distinct peaks can be clearly identified. The first peak occurs between 30 and 150 °C and is mainly associated with the dehydration of AFm phases, ettringite, and part of the C-S-H gel. The second peak occurs between 400 and 500 °C and corresponds to the dehydroxylation of calcium hydroxide (CH) [39].

As shown in Figure 13b, the incorporation of CNFs caused the C-S-H content in UHPC to first increase and then decrease. Compared with the control mixture, the C-S-H content increased by 6.5%, 16.0%, and 21.1% with the addition of 0.05%, 0.1%, and 0.2% CNFs, respectively. Among all mixtures, P2C0.2 exhibited the highest C-S-H content. This result indicates that an appropriate dosage of CNFs can effectively promote cement hydration and C-S-H formation in UHPC. The hydration-promoting effect of CNFs at low dosages can mainly be attributed to their high specific surface area and abundant surface hydroxyl groups. Well-dispersed CNFs can provide additional nucleation sites for hydration products and reduce the energy barrier required for nucleation. This promotes the precipitation and growth of C-S-H gel on both cement particles and CNF surfaces. In addition, the nanoscale filling effect of CNFs helps fill fine pores and interparticle voids in the hydrated paste. This contributes to the densification of both the cementitious matrix and the POMF–matrix interfacial region. CNFs also possess water absorption and retention capacities [40]. The locally stored water can be gradually released during hydration, which provides favorable conditions for continued hydration in UHPC with a low water-to-binder ratio. Therefore, the combined nucleation, nanoscale filling, and moisture-regulation effects of CNFs promote the formation of C-S-H. When the CNF dosage was further increased to 0.3%, the C-S-H content decreased to 21.86%. This value was approximately 11.4% lower than that of P2C0.2, but remained about 7.3% higher than that of P2C0. These results indicate that excessive CNFs weaken their beneficial effect on cement hydration. On the one hand, increasing the CNF dosage promotes fiber entanglement and agglomeration through hydrogen bonding. This creates locally heterogeneous regions and reduces the effective specific surface area and nucleation efficiency of CNFs. On the other hand, excessive CNFs can adsorb more mixing water and superplasticizer. This increases the viscosity and reduces the flowability of the fresh paste, thereby limiting the dispersion and wetting of cement particles.

#### 3.4.2. Microstructural Morphology Analysis

**Figure 14:**
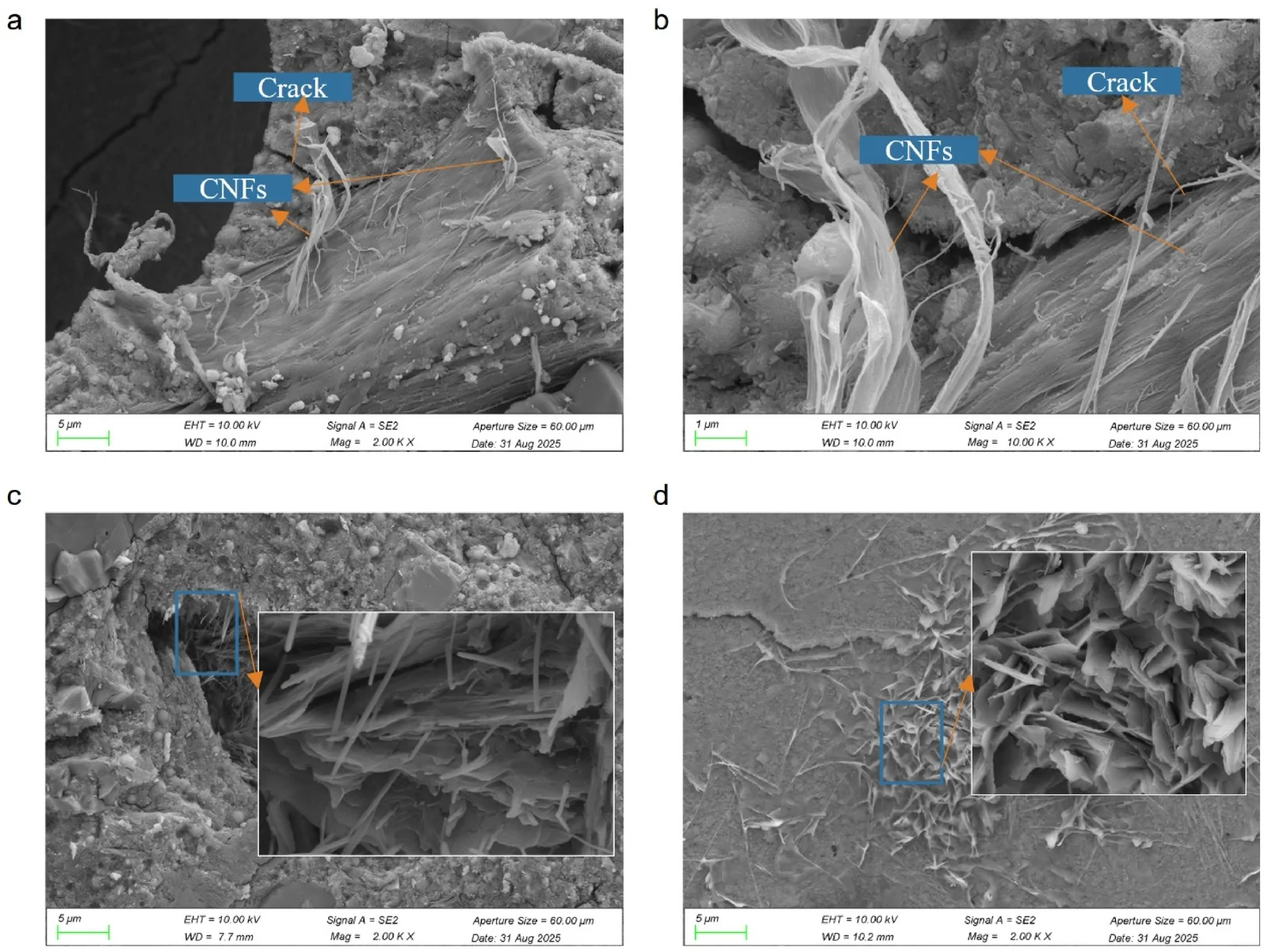
SEM images of the microstructural characteristics of POMF-reinforced UHPC and POMF–CNF-reinforced UHPC: (**a**) distribution of CNFs in P2C0.2; (**b**) nanoscale bridging of CNFs in P2C0.2; (**c**) morphology of hydration products in P2C0; and (**d**) morphology of hydration products in P2C0.2.

To further clarify the microstructural enhancement mechanism of CNFs in UHPC, SEM observations were conducted on specimens from different mixtures, as shown in Figure 14. The results indicate that CNFs improve the UHPC microstructure mainly through two mechanisms: nanoscale bridging and nucleation effects. As shown in Figure 14a,b, CNFs can be observed bridging adjacent hydration products in P2C0.2. Distinct nanoscale bridging structures are also present across local microcracks and micropores.

Due to their high aspect ratio and good flexibility, CNFs can connect relatively discrete hydration products and improve the structural continuity of the matrix. Under external loading, CNFs can transfer part of the applied stress through interfacial bonding and mechanical anchoring. This effect helps alleviate local stress concentrations and suppress the initiation and propagation of microcracks. Therefore, the nanoscale bridging effect of CNFs contributes to improved matrix integrity and enhanced mechanical performance of POMF–CNF-reinforced UHPC.

As shown in Figure 14c,d, the hydration products in P2C0 were relatively loosely distributed, and distinct interparticle pores were still observed in some regions. In contrast, P2C0.2 contained more abundant hydration products, with a greater degree of intergrowth and interconnection. As a result, the overall microstructure became denser. This indicates that an appropriate dosage of CNFs can promote the formation of hydration products and improve the microstructural packing of the UHPC matrix. This behavior is mainly associated with the high specific surface area and abundant surface hydroxyl groups of CNFs. CNFs can provide additional nucleation sites for hydration products and promote the precipitation and growth of C-S-H gel on their surfaces, thereby accelerating cement hydration. In addition, the nanoscale dimensions of CNFs allow them to fill fine pores between hydration products, reduce internal defects, and increase matrix densification. These observations are consistent with the TG analysis discussed above.

#### 3.4.3. Pore Structure Characteristics

**Figure 15:**
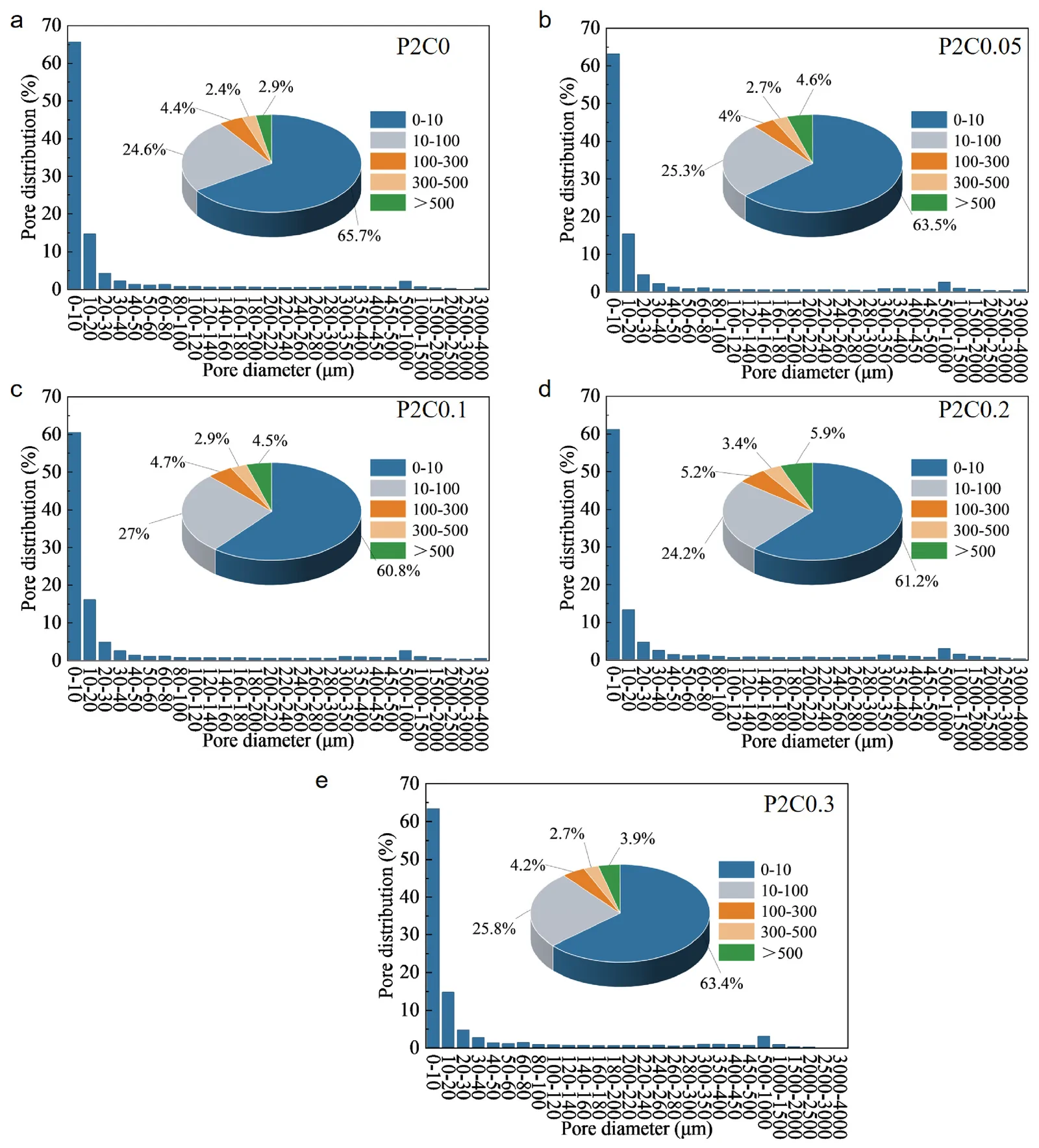
Pore size distribution of POMF-reinforced UHPC with different CNF contents. (**a**) P2C0; (**b**) P2C0.05; (**c**) P2C0.1; (**d**) P2C0.2; (**e**) P2C0.3.

The mechanical properties of UHPC are closely related to its pore structure. Figure 15 shows the pore size distributions of UHPC with different CNF dosages. For all mixtures, the pores were mainly concentrated within the 0–100 μm range, accounting for 85.47–90.17% of the total pore population. This indicates that small micrometer-scale pores were the dominant component of the UHPC pore system. For P2C0, the proportion of pores within the 0–100 μm range was 90.17%. After the incorporation of 0.05%, 0.1%, 0.2%, and 0.3% CNFs, this proportion decreased to 88.28%, 87.39%, 85.47%, and 89.14%, respectively. As the CNF dosage increased from 0 to 0.2%, the proportion of 0–100 μm pores gradually decreased and reached the lowest value in P2C0.2. When the CNF dosage was further increased to 0.3%, the proportion increased again. These results indicate that the incorporation of CNFs caused a redistribution of the pore size distribution in UHPC, with the most pronounced effect observed at a CNF dosage of 0.2%. Within the 0–100 μm range, pores smaller than 10 μm were dominant. Their proportion changed from 65.61% in P2C0 to 63.14%, 60.50%, 61.24%, and 63.39% after the incorporation of 0.05%, 0.1%, 0.2%, and 0.3% CNFs, respectively. Overall, the proportion first decreased and then increased. In contrast, the proportion of 10–100 μm pores varied only slightly and remained within 24.23–26.90%. Therefore, the variation in the total proportion of 0–100 μm pores after CNF incorporation was mainly associated with changes in the fraction of pores smaller than 10 μm, whereas the distribution of 10–100 μm pores remained relatively stable.

**Figure 16:**
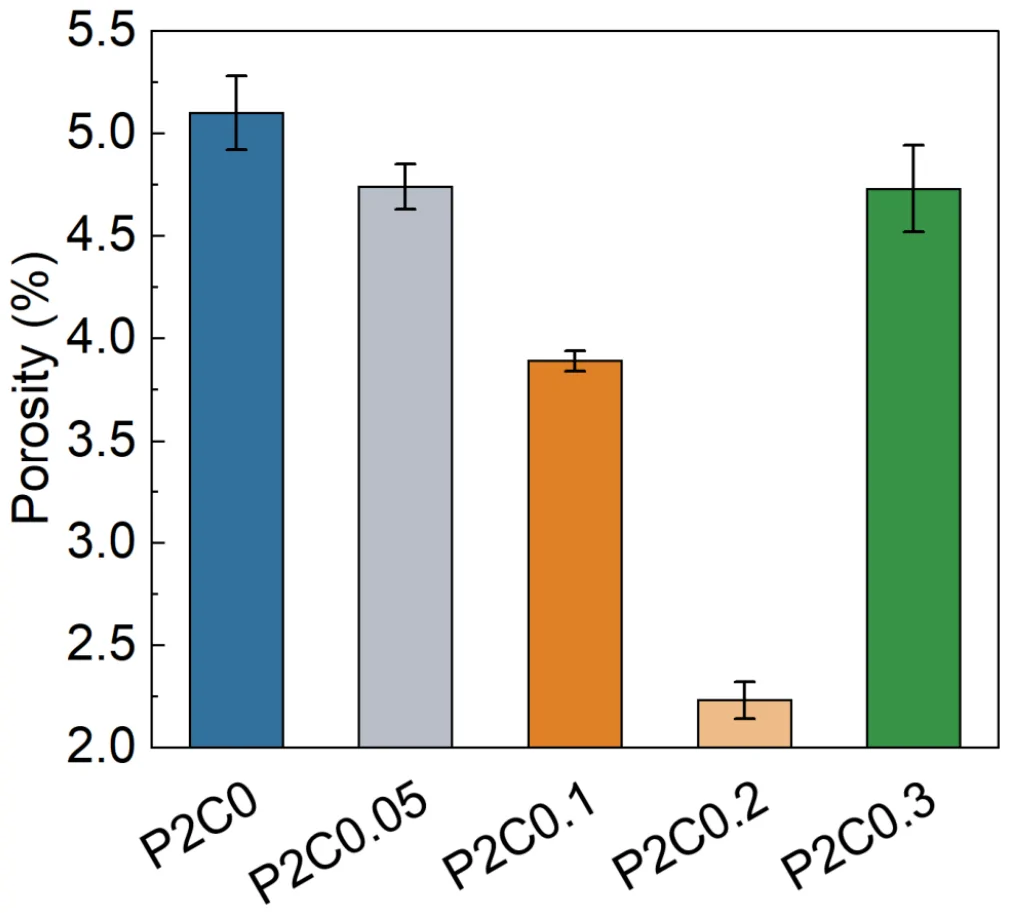
Porosity of UHPC specimens.

As shown in Figure 16, the total porosity of POMF-reinforced UHPC first decreased and then increased with increasing CNF dosage. The total porosity of P2C0 without CNFs was 5.10%. After the incorporation of 0.05 wt%, 0.1 wt%, and 0.2 wt% CNFs, the porosity decreased to 4.74%, 3.89%, and 2.23%, respectively. These values represent reductions of 7.1%, 23.7%, and 56.3% compared with P2C0. However, when the CNF dosage was further increased to 0.3 wt%, the total porosity increased slightly to 4.73%. This indicates that excessive CNFs are unfavorable for further pore structure refinement in UHPC. An appropriate dosage of CNFs can improve the pore structure of UHPC, mainly through nanoscale filling and nucleation effects.

### 3.5. Correlation Analysis

As discussed in Section 3.4, the incorporation of CNFs modifies the pore structure, hydration process, and microstructural characteristics of UHPC, thereby affecting its mechanical properties. To quantitatively evaluate the correlations between the microstructural parameters and mechanical properties, Pearson correlation analysis [41] was employed, as expressed in Equation (8) [42]. The correlation coefficient represents the covariance between two variables normalized by their respective standard deviations.

(8) $$r=\frac{\sum_{i=1}^{n}\left(X_{i}-\bar{X}\right)\left(Y_{i}-\bar{Y}\right)}{\sqrt{\sum_{i=1}^{n}{\left(X_{i}-\bar{X}\right)}^{2}}\sqrt{\sum_{i=1}^{n}{\left(Y_{i}-\bar{Y}\right)}^{2}}}$$

where r is the correlation coefficient. Xi and Yi are the values of the two variables for the iith sample. $\bar{X}$ and $\bar{Y}$ are the mean values of the two variables, and nn is the number of samples. A positive r indicates a positive correlation, while a negative r indicates a negative correlation. When r = 0, there is no linear correlation between the two variables. The closer r is to ±1, the stronger the correlation.

**Figure 17:**
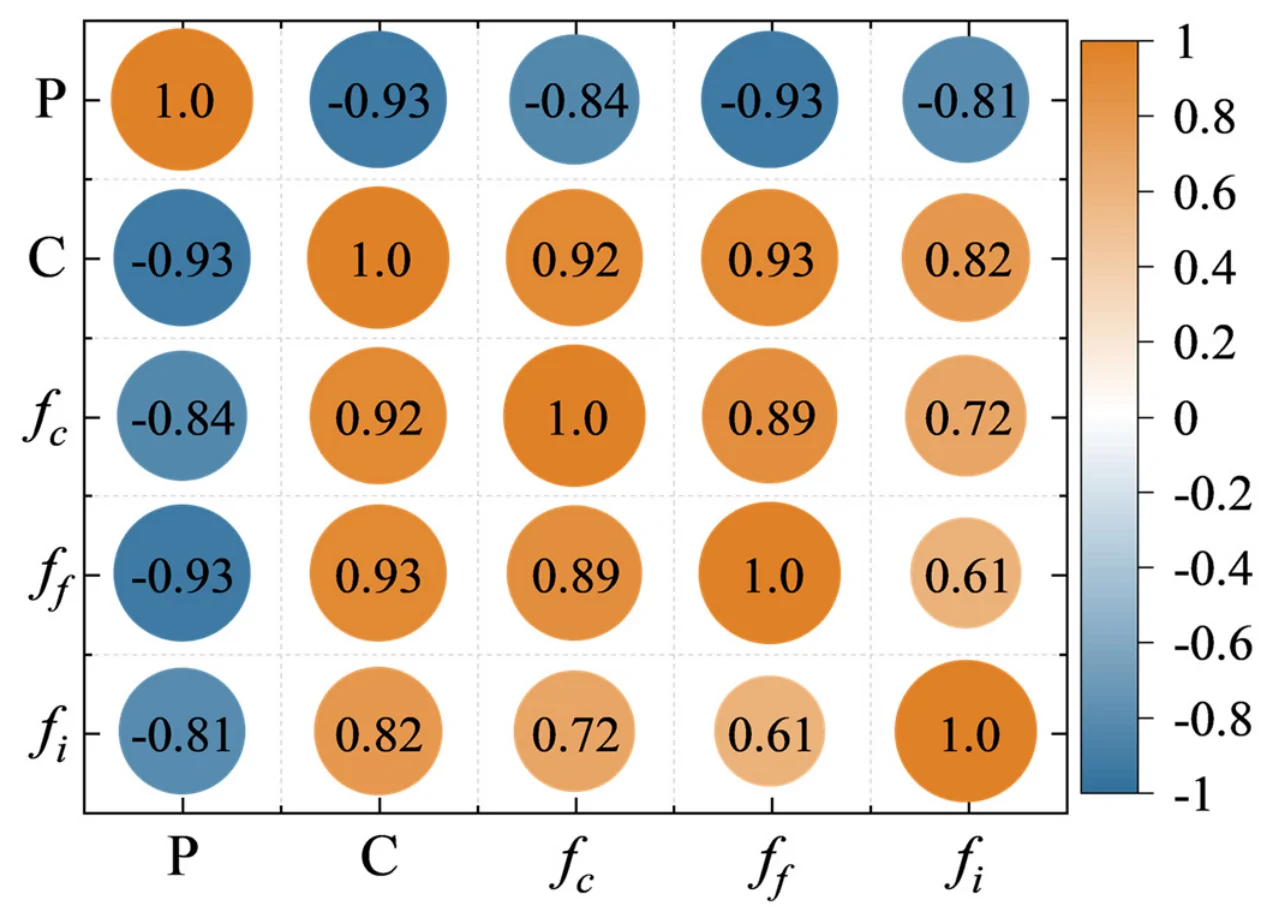
Pearson correlation heatmap of porosity, C-S-H content, and mechanical properties of CNF-modified POMF-reinforced UHPC.

Figure 17 shows the correlations among porosity (P), C-S-H content (C), and the mechanical properties of UHPC. Porosity exhibited a strong negative correlation with C-S-H content (r = −0.93), indicating a close relationship between increased hydration product formation and matrix densification. In addition, the correlation coefficients of P with compressive strength (*f_c_*), flexural tensile strength (*f_f_*), and impact strength (*f_i_*) were −0.84, −0.93, and −0.81, respectively. These results suggest that a reduction in pore defects generally benefits both the quasi-static and dynamic mechanical performance of UHPC.

In contrast, C-S-H content showed positive correlations with *f_c_*, *f_f_*, and *f_i_*, with correlation coefficients of 0.92, 0.93, and 0.82, respectively. This further indicates that the hydration-promoting effect of CNFs and the resulting matrix densification provide an important microstructural basis for the strength enhancement of POMF–CNF-reinforced UHPC.

Notably, the correlations of fi with P and C were weaker than those of *f_c_* and *f_f_*. In addition, the correlation coefficient between *f_f_* and *f_i_* was only 0.61. These results indicate that the dynamic impact resistance of UHPC is not governed solely by matrix densification or quasi-static strength. It is also strongly influenced by dynamic crack bridging by POMFs, stress transfer across the fiber–matrix interface, and energy dissipation through fiber pullout.

Therefore, CNFs improve the matrix not only by promoting C-S-H formation and refining the pore structure, but also by modifying the POMF–matrix interface. These effects may jointly influence the dynamic damage evolution and energy-dissipation behavior of POMF–CNF-reinforced UHPC.

## 4. Conclusions

In this study, a hierarchical reinforcement strategy combining POMFs and CNFs was proposed to improve the mechanical performance of UHPC. The influence of CNF incorporation on the mechanical properties and cross-scale strengthening mechanisms of POMF-reinforced UHPC was systematically investigated. The main conclusions are summarized as follows:

(1) POMFs make a limited contribution to the compressive strength of UHPC but improve its flexural load-bearing and post-cracking deformation capacities. The flexural strength increases by approximately 16.0%, while the failure mode changes from brittle to quasi-ductile. The incorporation of CNFs further enhances the quasi-static mechanical properties of POMF-reinforced UHPC. At a CNF content of 0.2 wt.%, the compressive and flexural strengths increase by 9.7% and 21.9%, respectively, compared with those of POMF-reinforced UHPC.
(2) POMF–CNF-reinforced UHPC exhibits a pronounced strain-rate strengthening effect, with both dynamic strength and impact toughness increasing with strain rate. Compared with POMF-reinforced UHPC, the addition of 0.2 wt.% CNFs increases the dynamic peak stress by approximately 32.1% and 15.2% at the lowest and highest strain rates, respectively, and provides the highest overall impact energy dissipation capacity. Excessive CNF addition, however, weakens the dynamic reinforcement effect.
(3) CNFs improve matrix compactness and the POM fiber–matrix interface through nucleation, nanoscale filling, and nanoscale bridging. At a CNF content of 0.2 wt.%, the C-S-H content increases by 21.1%, while the total porosity decreases by 56.3%. POMFs dissipate fracture energy through macroscopic crack bridging, interfacial slip, and fiber pullout. Together, these mechanisms establish a cross-scale reinforcement mechanism that extends from nanoscale defect control to macroscopic crack bridging.
(4) Porosity shows a strong negative correlation with compressive, flexural, and impact strengths, whereas C-S-H content is positively correlated with all three properties. Enhanced hydration and pore structure densification therefore provide an important microstructural basis for the improvement of UHPC mechanical performance by CNFs.

Future work will investigate broader POMF contents and loading conditions, develop strain-rate-dependent constitutive models for structural-scale simulations, and further assess the cost and environmental impacts of CNF incorporation to evaluate the engineering applicability and sustainability of POMF–CNF-reinforced UHPC.

## Figures and Tables

**Table 1:** Physical properties of the enhancement materials.

Appearance	Materials	Diameter	Length	Aspect Ratio	Elastic Modulus
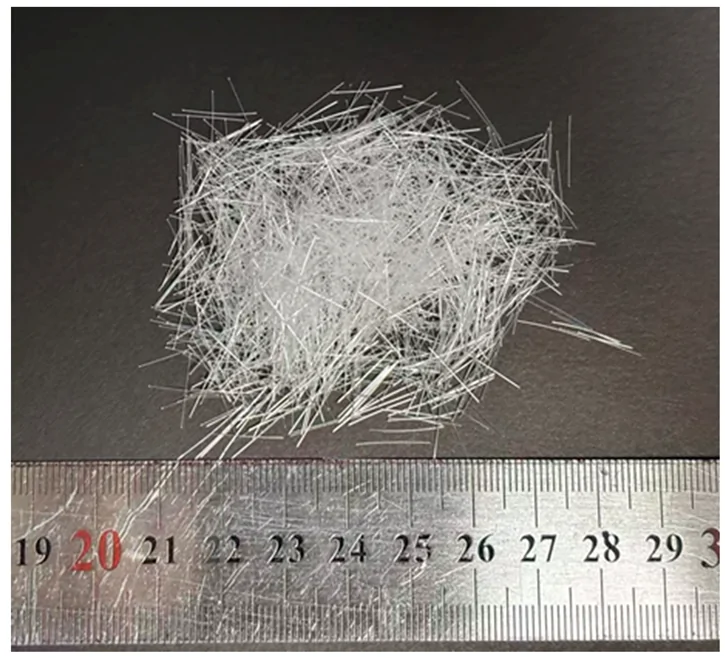	POMF	0.2 ± 0.02	12 mm	60	≥8.8 GPa
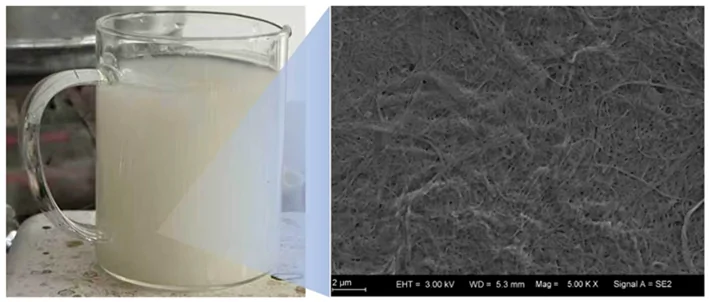	CNF	1–50 nm	1–30 μm	600–1000	135–150 GPa

**Table 2:** Mixture proportions for sample preparation (by weight).

Group	OPC	Silica Fume	Quartz Sand	SP	Cellulose Solid Content	POMF (kg/m^3^)
P0C0	1	0.25	1.1	0.015	-	-
P2C0	1	0.25	1.1	0.015	-	28.2
P2C0.05	1	0.25	1.1	0.015	0.0005	28.2
P2C0.1	1	0.25	1.1	0.015	0.001	28.2
P2C0.2	1	0.25	1.1	0.015	0.002	28.2
P2C0.3	1	0.25	1.1	0.015	0.003	28.2

## Data Availability

The original contributions presented in this study are included in the article. Further inquiries can be directed to the corresponding author.
